# Factors Associated with Treatment Compliance in Hypertension in Southwest Nigeria

**DOI:** 10.3329/jhpn.v29i6.9899

**Published:** 2011-12

**Authors:** Pauline E. Osamor, Bernard E. Owumi

**Affiliations:** ^1^Department of Sociology, Bowen University, Iwo, Osun State, Nigeria; ^2^Department of Sociology, University of Ibadan, Ibadan, Oyo State, Nigeria

**Keywords:** Adherence, Cardiovascular diseases, Community-based studies, Compliance, Cross-sectional studies, Hypertension, Nigeria

## Abstract

Hypertension is an important condition among adults, affecting nearly one billion people worldwide. Treatment with appropriate medication is a key factor in the control of hypertension and reduction in associated risk of complications. However, compliance with treatment is often sub-optimal, especially in developing countries. The present study investigated the factors associated with self-reported compliance among hypertensive subjects in a poor urban community in southwest Nigeria. This community-based cross-sectional study employed a survey of a convenience sample of 440 community residents with hypertension and eight focus-group discussions (FGDs) with a subset of the participants. Of the 440 hypertensiverespondents, 65.2% were women, about half had no formal education, and half were traders. Over 60% of the respondents sought care for their condition from the hospital while only 5% visited a chemist or a patent medicine vendor (PMV). Only 51% of the subjects reported high compliance. Factors associated with high self-reported compliance included: regular clinic attendance, not using non-Western prescription medication, and having social support from family members or friends who were concerned about the respondent's hypertension or who were helpful in reminding the respondent about taking medication. Beliefs about cause of hypertension were not associated with compliance. The findings of the FGDs showed that the respondents believed hypertension is curable with the use of both orthodox and traditional medicines and that a patient who ‘feels well’ could stop using antihypertensive medication. It is concluded that treatment compliance with antihypertensive medication remains sub-optimal in this Nigerian community. The factors associated with high self-reported compliance were identified. More research is needed to evaluate how such findings can be used for the control of hypertension at the community level.

## INTRODUCTION

Hypertension is an overwhelming global challenge, which ranks third as a means of reduction in disability-adjusted life-years (1). It affects approximately one billion people worldwide (4.5% of the current global burden of disease)—340 million of these in economically-developed and 340 million in economically-developing countries. Annually, it causes 7.1 million (or one-third of) global preventable premature deaths (2,3). Due to the fact that hypertension is one of the most important modifiable risk factors for cardiovascular diseases (4), treatment that commences once it is recognized reduces the cardiovascular risk of the individual. Therefore, access to treatment with antihypertensive medication and compliance with treatment are key factors in the control of hypertension. Hypertension, the leading cause of mortality and the third largest cause of disability, is poorly controlled worldwide. It is estimated that almost one-half of patients drop out entirely from treatment within one year (5). The failure to control hypertension takes an unacceptable toll on patients and their families. In addition to the personal cost, to the individual patient, uncontrolled hypertension creates huge, avoidable economic burdens when viewed in terms of the general population.

The total number of estimated deaths resulting from all types of cardiovascular diseases and hypertensive heart disease recorded for Nigeria in 2004 by the World Health Organization (WHO) was 201,500 and 10,700 respectively (6) and placed Nigeria in the 16th position globally. Although these numbers are low compared to 922,700 and 229,000 deaths reported for the USA and the United Kingdom respectively, it is clear that there is a growing health problem that requires an intervention. Uncontrolled blood pressure has been demonstrated to be a major risk factor contributing to more than 500,000 cases of stroke and one million myocardial infarction cases reported each year in the United States alone (7). An estimated 14.55 million people, worldwide, aged 30-80 years, were reported to have died as a result of hypertension-related conditions in 2005, of which 7.03% were reported for sub-Saharan Africa (8).

Traditionally, the term compliance has been employed to mean the extent to which the patient, when taking a drug, complies with the clinician's advice and follows the regimen (9). Compliance with treatment is defined and characterized when medical or health advice coincides with the individual's behaviour with regard to the use of medication, recommended changes in lifestyle, and attendance to medical appointments (10). Poor compliance with treatment is the most important cause of uncontrolled blood pressure (11). Results of studies in the United States revealed that long-term compliance with treatment is always a problem in any chronic disease condition, and hypertension is no exception. More than 50% of patients in the United States, who were prescribed antihypertensive medications actually discontinued therapy within 12 months (12). A common reasongiven for stopping medication relates to adverse effects, although the patient's knowledge about the disease, attitudes regarding treatment of an often asymptomatic condition, and personal health beliefs, together with cost of medications and availability of healthcare, are major contributors (12).

Multiple factors contribute to poor compliance with long-term antihypertensive therapy. Many patients have negative attitudes towards taking medication, especially if they ‘feel well’ (13). According to Jadelson *et al*., the major reasons for non-complianceare multi-factorial and range from lack of adequate guidance to socioeconomic status (14). Although the socioeconomic status has not consistently been found to be an independent predictor of compliance low socioeconomic status may put patients in developing countries in the position of having to choose between competing priorities (15). Such priorities include demands to direct the limited resource available to meet the needs of other family members, such as children or parents, for whom they care. Some factors reported to have a significant effect on compliance are: poor socioeconomic status (poverty), low level of education, unemployment, lack of effective social support networks, unstable living conditions, long distance from treatment centre, high cost of transport, cultural and lay beliefs about illness and treatment, and forgetfulness (16).

The present study describes treatment-compliance patterns among hypertensive subjects in a Nigerian community and investigates the factors associated with good compliance, including demographic factors, beliefs about hypertension, and the availability of social support.

## MATERIALS AND METHODS

### Study design and recruitment of sample

The study was conducted in the Idikan community, Ibadan, a city in the southwestern Nigeria, as part of a larger community-based study of the sociological aspects of hypertension. Idikan is located in the indigenous part of the city of Ibadan. Idikan is a densely-populated urban community within Ibadan city, with a population of 7,883 (17). Health facilities in the community include an outreach clinic run by the Department of Community Health of the University of Ibadan, a small dental clinic run by the Dental Centre of the University College Hospital (UCH), and private clinics. There are also registered patent medicine stores (pharmacies) and traditional healing homes in the area, all of which are accessible to members of the community.

The study consisted of two components: a quantitative study using a community-based survey of hypertensive subjects and a qualitative study using focus-group discussions (FGDs) on a subset of the participants. The subjects for the quantitative study were adult (aged above 25 years) residents of Idikan who are known to have hypertension. Previous studies in the community had conducted household screening for hypertension, which facilitated the identification of hypertensive subjects in the community. The subjects for this study were selected from a list of known hypertensive subjects residing in the community that was prepared for one such previous hypertension study (18) and was updated for the present study during visits to the home. Four hundred and forty hypertensive subjects were enrolled using a consecutive sampling method. The inclusion criteria were: (a) adults aged over 25 years, (b) having diagnosed hypertension by blood-pressure measurements, and (c) awareness of the hypertension status. The only exclusion criteria were refusal to participate and recent (less than three years) diagnosis of hypertension since the study required respondents to have experience living with hypertension to be able to answer the questions. After obtaining informed consent, the subjects were administered a semi-structured questionnaire that had items on several issues, including healthcare-seeking for their hypertension, their beliefs about hypertension, compliance with treatment, and availability of social support (from family and friends).

The goal of the FGDs was to capture in-depth information that is complementary to the quantitative study (survey). This instrument guide had questions on knowledge, beliefs, perceptions, care-seeking behaviour, other experiences, and compliance with treatment for hypertension. Specific probes were included on the reasons (the ‘why’ and ‘how’) for the respondent's beliefs, attitude, and actions regarding hypertension they have. Eight FGDs were carried out. A purposive sampling technique was used for selecting participants for group discussion, and discussants were homogeneous in characteristics within each group. There were four groups of males and four groups of females. Each group comprised 6-8 discussants. The inclusion criteria were individuals who were aged 45-60 years. They would have also been diagnosed of high blood pressure for 3-7 years. This allowed for having experiences living with hypertension while minimizing the forgetfulness of care-seeking over time. The key variable reflected in the composition of the focus groups was gender (male vs female). Gender is important because it reflects a major determinant of life experiences of people in the community and to ensure that the discussion and interaction among the participants in the FGDs is free and open. This provided relatively-homogenous focus groups that facilitated free and open discussion on which theamount of information that was elicited depends.

### Analysis of data

Management and analysis of the survey questionnaire data were done using the SPSS software (version 14) (SPSS Inc., Chicago, USA). Frequencies of the responses to the questions were computed and presented as percentages. The association between categorical variables was tested using the chi-square test. Compliance was defined using the question on how frequently people missed taking their medication. Compliance as a variable was defined and used in two ways. First, compliance was scaled as: ‘high compliance’ (where the respondent ‘never misses’ or ‘rarely misses’ to take his/her medication doses); ‘medium’ compliance (where the respondent ‘sometimes’ misses taking medication); and ‘low compliance’ (where the respondent ‘regularly’ or ‘fairly regularly’ misses to take his/her medication. These variables were used for identifying the factors associated with compliance in general. Second, since the desired goal of treatment for hypertension is that the patient complies with taking medication for controlling his/her high blood pressure, ‘high compliance’ (where the respondent never misses or rarely misses to take his/her medication doses) was used for identifying the variables associated with this goal of therapy.

Qualitative data were transcribed as soon as possible after each FGD session. The first author analyzed the qualitative data, reading through all notes and transcripts of the FGDs and identifying emerging themes relating to treatment compliance. Computer-assisted qualitative data analysis (CAQDAS) was also done using the *ATLAS*.ti software.

### Ethical approval

Ethical approval for the study protocol was obtained from the Institutional Review Committee of the University of Ibadan/University College Hospital, and written informed consent was obtained from all the participants.

## RESULTS

### Sociodemographic characteristics

Of the 440 survey respondents, 65.2% were women. About half (51.1%) of the respondents had no formal education. In terms of occupation, 50% were traders, and about 26% were retired or not working while 11% were artisans. The respondents were aged 25-90 years. Their mean age was 60 [standard deviation (SD) 12] years. Dividing the age distribution of the respondents into 10-year bands, the peak age-categories were 46-55 years and 56-65 years comprising 29.3% and 29.1% of the respondents respectively. There was no significant relationship between the gender of the respondents and their age distribution. The large majority (71%) ofthe respondents were married (Table 1). A large proportion (63.4%) of the respondents sought care for their condition from a hospital (the University College Hospital, the community health centre, or a private hospital) while 5% visited a chemist or a patent medicine vendor. About 9.5% of the respondents who visited the hospital also used traditional medicine while 7.3% visited the chemist and used traditional medicine. None visited a traditional healer exclusively.

**Table 1.** Demographic characteristics of survey respondents (n=440)

Characteristics	No.	%
Religion		
Islam	270	61.4
Christianity	169	38.4
Traditional	1	0.2
Ethnic group		
Yoruba	434	98.6
Ibo	5	1.1
Isoko	1	0.2
Level of education		
No formal education	225	51.1
Primary	86	19.5
Secondary	49	11.1
Post-secondary	77	17.5
Others (Arabic school)	3	0.7
Occupation		
Trading	220	50.0
Artisanry	49	11.1
Teaching/civil service	43	9.8
Not working (retired)	113	25.7
Religious teaching	15	3.4

### Treatment-compliance patterns among respondents

The findings of the survey showed that 77.5% of the respondents complied with keeping their follow-up clinic appointments every time. Over one-half (50.7%) of the respondents had high self-reported compliance with treatment as they claimed to be taking their medication regularly whereas 41.5% had poor self-reported compliance at different levels ranging from regularly missing to take their medication to rarely taking their medication. Forty-six percent had taken their medication on the day of the study.

**Table 2.** Relationship between beliefs about cause of hypertension and high self-reported compliance

Perceived cause of hypertension	Response	High self-reported compliance		
		No. (%)	χ^2^	p value
Anxiety	Yes	88 (56.1)	0.559	0.455
	No	169 (59.7)		
Stress	Yes	69 (62.1)	0.861	0.354
	No	188 (57.1)		
Do not know	Yes	53 (68.8)	4.173	0.041
	No	204 (56.2)		

Eleven percent of the respondents who were non-compliant to medication felt better and, therefore, had no need to continue taking their medication. Other factors included: forgetfulness (8.4%), lack of funds to purchase drugs (6.8%), side-effects of drugs (6.1%), and having a busy schedule but limited medication (3.6%). Only 0.5% said that they were tired of taking drugs. When the respondents perceived forgetting to take medication and side-effects of treatment as problematic, they were less likely to comply with treatment.

### Factors associated with good compliance

Educational status and religion were two factors that often influenced knowledge, beliefs, attitudes, and practices relating to health and other domains of life. The findings showed that high self-reported compliance was not associated with the religion professed by the respondent. Almost equal percentages of Muslims and Christians (57.8% vs 59.2%) showed high self-reported compliance (χ^2^=0.797, p=0.671). Concerning education, there was no clear-cut trend with high self-reported compliance (χ^2^=6.683, p=0.245), although those with primary education showed a higher frequency of high self-reported compliance when compared with respondents with other categories of educational levels. Beliefs about the perceived cause of hypertension were not significantly associated with treatment compliance.

There was no association between believing that anxiety or stress is a cause of hypertension and high self-reported compliance. On the other hand, a higher percentage of those whose response to the question on the cause of hypertension was ‘Do not know’ reported high compliance compared to those who professed to know the cause of hypertension (68.8% vs 56.2%) (Table 2). Keeping regular clinic appointments and use/non-use of non-Western medication were significantly (p<0.0001) associated with high self-reported compliance. Table 3 shows that the respondents who attended clinic appointments ‘every time’ showed the highest percentage (67.5%) of high self-reported compliance compared to those who attended clinic appointments ‘most times’ (42.1%) or less frequently. In other words, regularly keeping clinic appointments was positively associated with high self-reported compliance. In addition, the use of non-Western medication was also significantly related to high self-reported compliance: a significantly lower percentage of those who used non-Western medication showed higher self-reported compliance than those who did not (45.3% vs 63.8%, p<0.001).

**Table 3.** Association between practices and high self-reported compliance

Practice	Response	High self-reported compliance		
		No. (%)	χ^2^	p value
Keeping regular clinic appointments	Every time	230 (67.5)	66.572	<0.0001
	Most times	16 (42.1)		
	Sometimes	3 (8.8)		
	Miss most	1 (6.7)		
	Rarely keep	7 (58.3)		

**Table 4.** Association between beliefs about hypertension and treatment compliance

Belief about hypertension	Response	Compliance (%)			χ^2^	p value
		High	Medium	Low		
Hypertension is preventable	Yes	57.0	13.7	29.4	8.272	0.082
	No	42.1	31.6	26.3		
	Do not know	64.1	15.6	20.3		
Hypertension is curable	Yes	55.6	15.8	28.6	7.744	0.101
	No	51.6	16.1	32.2		
	Do not know	71.3	11.5	17.2		
Hypertension is serious	Yes	58.2	14.7	27.1	1.778	0.777
	No	50.0	25.0	25.0		
	Do not know	65.4	15.4	19.2		
Hypertension can lead to complications	Yes	54.5	14.7	26.8	2.106	0.716
	No	80.0	20.0	0.0		
	Do not know	55.0	17.5	27.5		
Hypertension-related complications can be prevented	Yes	50.0	20.7	29.3	3.774	0.437
	No	60.3	13.6	26.2		
	Do not know	61.0	14.6	24.4		
A person on treatment can stop taking medication after sometime	Yes	49.5	28.3	22.2	19.658	0.001
	No	60.2	10.7	29.1		
	Do not know	66.7	14.3	19.1		

The relationship between beliefs about hypertension may be important to know how well they comply with treatment. These beliefs include the notions that hypertension is preventable, is curable, is a serious condition, and can lead to complications. For these analyses, their relationships with compliance in general and with high self-reported compliance (as defined above) were explored. Of the other beliefs about hypertension studied, only the belief that hypertension medication can be stopped after a while was significantly (p=0.001) associated with compliance in general (Table 4).

Going on to explore the factors associated with high self-reported compliance, the belief that hypertension was curable was significantly (p=0.023) associated with high self-reported compliance, with 55.6% of those who said ‘Yes’ versus 51.6% of those who answered ‘No’ and 71.3% of those who said ‘Do not know 'showing high self-reported compliance. Also, a lower proportion of those who believed that hypertension medication can be stopped after a while showed high self-reported compliance (49.5% vs 60.2% of those who answered ‘No’ and 66.7% of those who did not know) but this was not significant (p=-0.090) (Table 5).

Several potential factors that could affect compliance with treatment, including having a family member with hypertension or who has experienced complications from hypertension, got support from family members and friends were explored in the present project. Most of these factors, including having a family member with hypertension and support from family members and friends, were associated with compliance in general. As shown in Table 6, having family members with hypertension and having a family member who has suffered complications were not associated with high self-reported compliance. On the other hand, having friends who were concerned about the respondent's hypertension or who were helpful in reminding the respondent about taking medication was associated with high self-reported compliance.

**Table 5.** Relationships between other beliefs about hypertension and high self-reported compliance

Belief about hypertension	High self-reported compliance		
	%	χ^2^	p value
Hypertension is preventable	57.0	4.004	0.135
Hypertension is curable	55.6	7.561	0.023
Hypertension is serious	58.2	0.877	0.645
Hypertension can lead to complications	58.5	1.152	0.562
Hypertension-related complications can be prevented	50.0	2.941	0.230
A person on treatment can stop taking medication after some time	49.5	4.812	0.090

**Table 6.** Relationships between other factors and treatment compliance

Variable	Response	Compliance (%)			χ^2^	p value
		High	Medium	Low		
Has a family member with hypertension	Yes	49.3	23.3	27.4	10.146	0.038
	No	61.7	13.5	24.9		
	Do not know	45.5	12.1	42.4		
Has a family member who has serious health problems due to hypertension	Yes	36.4	21.2	42.4	12.060	0.017
	No	61.2	14.9	23.9		
	Do not know	48.4	9.7	41.9		
Family members concerned about respondent's hypertension	Not very concerned	47.6	28.6	23.8	10.857	0.093
	Very concerned	50.6	23.5	25.9		
	Extremely concerned	61.1	11.9	27.1		
	Do not know	60.0	20.0	20.0		
Family members helpful in reminding about medication	Not very helpful	54.6	18.2	27.3	13.579	0.035
	Very helpful	48.3	27.0	24.7		
	Extremely helpful	61.5	11.5	27.0		
	Do not know	57.1	14.3	28.6		
Friends concerned about respondent's hypertension	Not very concerned	49.0	10.8	40.2	62.203	<0.0001
	Very concerned	50.9	27.6	21.6		
	Extremely concerned	80.3	10.2	9.5		
	Do not know	33.3	0.0	66.7		
Friends helpful in reminding about medication	Not very helpful	49.2	12.3	38.5	62.204	<0.0001
	Very helpful	44.0	31.9	24.2		
	Extremely helpful	79.3	8.0	12.7		
	Do not know	50.0	25.0	25.0		

The findings of the FGD on issues of drug compliance showed that, in general, the respondents perceived antihypertensive medications to be necessary but indicated those factors that hindered compliance with treatment. The major factors identified in the FGDs included: (a) Costs: medications were expensive compared to income, and some participants could only buy the quantity of medication they could afford instead of the full prescription (for example, two weeks instead of four weeks). This was the factor that was most emphasized; (b) Since having high blood pressure did not make them feel sick, respondents may question the rationale for daily treatment without an end in sight. Even after they became aware of their hypertension and had been on treatment for a while, this rationale persisted, and they often took their medication sporadically and, in some cases, stopped taking their medication after some time; and (c) Difficulty with the idea and practice of taking a medication every day for life. First, the idea of taking a medication daily for life meant that there was no cure, and in any case, many did not see the reason for continuing to take medication when they felt better or their blood pressure was controlled. Second, the participants said that they sometimes forgot to take their medication. These were the three major themes that emerged from the FGDs. Discussants 'quotes that illustrate these obstacles are shown in the Figure.

**Fig.**
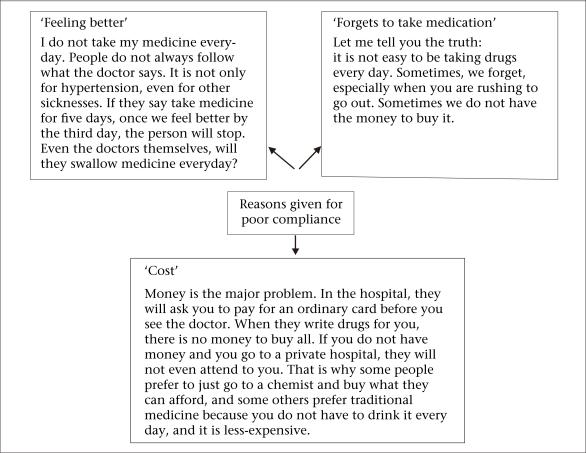
Reasons given for poor compliance in focus groups

## DISCUSSION

Compliance with treatment is a very important issue in the successful control of hypertension andprevention of complications. In prescribing medication, compliance usually means “the extent to which the patient takes the medication as prescribed” (19). A number of factors influence compliance. The common belief that patients are solely responsible for taking their treatment is misleading and often reflects a misunderstanding of how other factors affect people's behaviour and capacity to adhere to their treatment.

Belief in the necessity of antihypertensive medication was high among the respondents, and the majority believed that it was necessary to take antihypertensive medication even if one does not feel sick. About 19% believed that one should only take medication when there are symptoms and had strong concerns about the potential adverse effects of taking medication every day or did not see the need for taking medication when one is not feeling ill. This finding also provides a preliminary insight into the mechanism by which beliefs relating to medication might influence compliance. Some of these findings were similar to those reported in previous studies (20,21). Familoni *et al*., in a 2004 study in Nigeria, reported that only about one-third of patients knew that hypertension should ideally be treated for life, and 58.3% believed that antihypertensive drugs should be used only where there are ‘symptoms’ while the remaining 6.3% believed that the treatment should be for a period of time and not for life (22). Although it has been suggested that it is sometimes possible to withdraw drug therapy and continue lifestyle-modification after several years (23), the consensus is that almost all who are hypertensive before treatment will become hypertensive again if treatment is stopped. This practice has sometimes resulted in disastrous consequences.

There are many concepts that refer to compliance, for example ‘compliance’, ‘adherence’, ‘commitment’, and ‘concordance’. According to Kontz, the most important thing is how the content of the concept is defined (24). A major factor accounting for the inadequate treatment of hypertension is poor compliance. The findings of this study revealed that almost half of the respondents reported high compliance with treatment with drug, and 86% claimed high compliance with keeping their appointments with doctors. Reasons for compliance with treatment include fear of the complications of hypertension and the desire to control blood pressure. Benson, in 2002, reported that patients comply with medication regimen for various reasons, including perceived benefits of medication, fear of complications associated with hypertension, and feeling better on medication (21). The latter reason is contrary to the generally-held belief among physicians that hypertension is a largely asymptomatic disease (25).

Interestingly, about one-half of the study respondents were non-compliant to their medication. The results of the FGDs suggest that the decision to stop using antihypertensive medication is influenced by the beliefs the respondents hold concerning these medications. The identification of the factors determining non-compliance and a better knowledge about them could allow the implementation of measures that could enable their correction and providing the adequate control of blood-pressure levels. In this study, reasons for non-compliance with medication are multifactorial and range from low level of knowledge regarding hypertension as a disease and lack of adequate guidance to socioeconomic aspects. The identification of side-effects of treatment used represents another cause of non-compliance with treatment. This finding lays credence to the submission of Hyman and Pavlik that a primary reason given for stopping medication relates to adverse effects, although the patient's knowledge about the disease, attitudes towards treatment of an often asymptomatic condition, and personal health beliefs, together with cost of medications and the availability of healthcare, are major contributors (12). One central theme that runs through the data in this study is the issue of socioeconomic status of the respondents. Financial hardship is a significant barrier to complying with treatment and is a contributory factor to non-compliance. If people are hungry, nothing matters, except food. People either take medication very late when they have had something to eat or forget about it while trying to deal with other problems of poverty. This finding corroborates the observed association among poor compliance, ignorance, and lack of funds for the purchase of drugs reported by Isezuo and Opara (26).

While stress and anxiety were the two most common perceived causes of hypertension among the respondents, the findings showed that those who held these beliefs did not show better compliance than others. However, those who did not know the cause of hypertension showed better compliance with medication than others. This implies that not having preconceived ideas about the cause of hypertension made it more likely that the respondents would comply with treatment. This observation was also common to other beliefs about hypertension (being curable, preventable, serious, leading to complications, and so on) where those who responded ‘Do not know’ showed a tendency to better compliance. Other studies that have investigated the relationship between beliefs and compliance reported that patient's belief and lack of knowledge, along with other factors, influenced their response to treatment (27,28).

Social support networks are important in the long-term management of chronic conditions, such as hypertension, which requires a radical and life-long change in the lifestyle of the affected person. In the present study, those who had support from friends or family members (concerned about their illness, giving reminders about medication) had better compliance with treatment than those who did not, although this difference was the greatest for those who had the support of friends. This is an important finding and is consistent with what has been reported for multiple chronic diseases in several parts of the world (29). Interestingly, the evidence from the present study shows that support from friends is a stronger factor influencing high self-reported compliance than support from family members. This may be a reflection of the fact that most people in the community (and in cities in general) see, talk, and interact more with their friends than with their family members who do not live nearby. Another explanation may be that those with hypertension are more likely to discuss their health problems with their friends than with their family members, thereby inadvertently limiting the support they could receive from the latter.

### Limitation

A potential limitation of this study is that compliance was measured using self-report and, therefore, suffers from the problems of recall. Inclusion of methods, such as pill-counts or more sophisticated electronic methods, may have helped more accurately assess compliance. However, this was not feasible within the context of a community-based study. Nonetheless, the study provides useful data in this important area of compliance to therapy for a non-communicable disease.

### Conclusions

It is concluded that the control of hypertension is still sub-optimal in this community, with only one-half of the affected persons reporting high compliance with treatment. The factors associated with high self-reported compliance included: regular clinic attendance, not using non-Western prescription medication, and having social support from family members or friends who were concerned about the respondent's hypertension or who were helpful in reminding the respondent about taking medication. Most respondents believed that hypertension is curable with the use of both orthodox and traditional medicines and that a patient who ‘feels well’ could stop using antihypertensive medication. On the other hand, specific beliefs about the cause of hypertension were not associated with compliance.

It is recommended that similar research be conducted in developing countries on factors affecting compliance in hypertension and similar diseases that require life-long medication for control. Furthermore, studies are needed on how such findings can be used for guiding local hypertension-control efforts.

## ACKNOWLEDGEMENTS

The authors thank the study participants and leaders of the Idikan community, Ibadan. The input of Dr. Adebowale Adeyemo of the Center for Research in Genomics and Global Health of the National Institutes of Health, Bethesda, MD, USA, at various stages of the project is hereby acknowledged.
